# Genomic, transcriptomic, and metabolomic profiles of hiPSC-derived dopamine neurons from clinically discordant brothers with identical *PRKN* deletions

**DOI:** 10.1038/s41531-022-00346-3

**Published:** 2022-06-29

**Authors:** Holly N. Cukier, Hyunjin Kim, Anthony J. Griswold, Simona G. Codreanu, Lisa M. Prince, Stacy D. Sherrod, John A. McLean, Derek M. Dykxhoorn, Kevin C. Ess, Peter Hedera, Aaron B. Bowman, M. Diana Neely

**Affiliations:** 1 John P. Hussman Institute for Human Genomics, University of Miami Miller School of Medicine, Miami, FL USA; 2 Department of Neurology, University of Miami Miller School of Medicine, Miami, FL USA; 3 John T. Macdonald Foundation Department of Human Genetics, University of Miami Miller School of Medicine, Miami, FL USA; 4 School of Health Sciences, Purdue University, West Lafayette, Indiana, IN USA; 5 Center for Innovative Technology, Vanderbilt University, Nashville, TN USA; 6 Department of Chemistry, Vanderbilt University, Nashville, TN USA; 7 Department of Neurology, Vanderbilt University Medical Center, Nashville, TN USA; 8 Department of Pediatrics, Vanderbilt University Medical Center, Nashville, TN USA; 9 Department of Neurology, University of Louisville, Louisville, KY USA

**Keywords:** Cellular neuroscience, Parkinson's disease, Stem-cell differentiation

## Abstract

We previously reported on two brothers who carry identical compound heterozygous *PRKN* mutations yet present with significantly different Parkinson’s Disease (PD) clinical phenotypes. Juvenile cases demonstrate that PD is not necessarily an aging-associated disease. Indeed, evidence for a developmental component to PD pathogenesis is accumulating. Thus, we hypothesized that the presence of additional genetic modifiers, including genetic loci relevant to mesencephalic dopamine neuron development, could potentially contribute to the different clinical manifestations of the two brothers. We differentiated human-induced pluripotent stem cells (hiPSCs) derived from the two brothers into mesencephalic neural precursor cells and early postmitotic dopaminergic neurons and performed wholeexome sequencing and transcriptomic and metabolomic analyses. No significant differences in the expression of canonical dopamine neuron differentiation markers were observed. Yet our transcriptomic analysis revealed a significant downregulation of the expression of three neurodevelopmentally relevant cell adhesion molecules, *CNTN6*, *CNTN4* and *CHL1*, in the cultures of the more severely affected brother. In addition, several *HLA* genes, known to play a role in neurodevelopment, were differentially regulated. The expression of *EN2*, a transcription factor crucial for mesencephalic dopamine neuron development, was also differentially regulated. We further identified differences in cellular processes relevant to dopamine metabolism. Lastly, wholeexome sequencing, transcriptomics and metabolomics data all revealed differences in glutathione (GSH) homeostasis, the dysregulation of which has been previously associated with PD. In summary, we identified genetic differences which could potentially, at least partially, contribute to the discordant clinical PD presentation of the two brothers.

## Introduction

First described over 200 years ago, Parkinson’s Disease (PD) is the second most common neurodegenerative disorder, a progressive disease with a characteristic loss of substantia nigra dopaminergic neurons. PD is believed to result from the interaction of genetic and environmental factors^1^. To date, more than 20 genes have been associated with increased PD risk and genome wide association studies (GWAS) have further implicated at least 90 risk variants^2,3^. While the majority of patients suffer from idiopathic PD, meaning no obvious causal genetic variant has been identified, there are indications that at least some forms of idiopathic PD have a complex genetic architecture^4^. About 5–10% of PD cases are familial and associated with known genetic mutations showing autosomal dominant or recessive inheritance patterns^3,5,6^.

Mutations in the *parkin RBR E3 ubiquitin-protein ligase* (*PRKN*, also known as *PARK2*) gene are the most common cause of recessively inherited and early-onset PD^7–9^. Over 100 pathogenic *PRKN* mutations have been described including missense mutations, exon rearrangements and copy number variations^10–14^. Age of onset and disease progression in patients with *PRKN* mutations can vary greatly^10,15–17^ and the marked variability of clinical phenotypes, even among family members carrying the same *PRKN* mutations, suggests that additional factors play a role in disease pathogenesis^17–20^.

The occurrence of juvenile cases demonstrates that PD is not necessarily an aging-associated disease. Indeed, evidence is accumulating that there is a developmental component to PD pathogenesis. Minor developmental defects could result in changes in the number of dopamine neurons, their connectivity, insult tolerance or compensatory mechanisms of the dopaminergic circuitry^21–25^. The developmental hypothesis is supported not only by the occurrence of juvenile PD cases, but also by the examination of PD postmortem brain samples^26–28^ and studies in animal models^22,25,29–32^. Indeed, developmental defects relevant to PD have been described for human neuroprogenitor cells as well as postmitotic neurons^4,21,26,33,34^.

We have previously reported on two brothers (PM and SM) of European ancestry who carry identical compound heterozygous *PRKN* mutations but present with significant phenotypic discordance with respect to PD onset, disease progression, and clinical symptoms^20^. The older brother, PM, presented with intermittent bilateral upper extremity action tremors by age 18 and developed marked parkinsonism in his mid-20s. His younger brother, SM, presented with minimal hypomimia and exercise-induced foot dystonia at age 39, but when examined at age 47 he did not meet the UK Brain Bank Criteria for PD^35^. This difference in clinical presentation between these two brothers is interesting given that they carry identical deletions in *PRKN*, grew up in the same household, and have spent most of their lives in the same geographical area, suggesting similar exposure to potential environmental risk factors. The divergence in their clinical presentation led us to hypothesize the presence of neurodevelopmentally relevant genetic differences that could potentially play a role in the divergence of disease onset and progression in these two brothers. To test our hypothesis, we compared whole exome sequencing, transcriptomic data, and metabolomic profiling of mitotically active mesencephalic neural precursor cells (floor plate cells) and early postmitotic dopaminergic neurons differentiated from human induced pluripotent stem cells (hiPSCs) from the two brothers.

## Results

### Validation and characterization of PM and SM hiPSCs

**Fig. 1:**
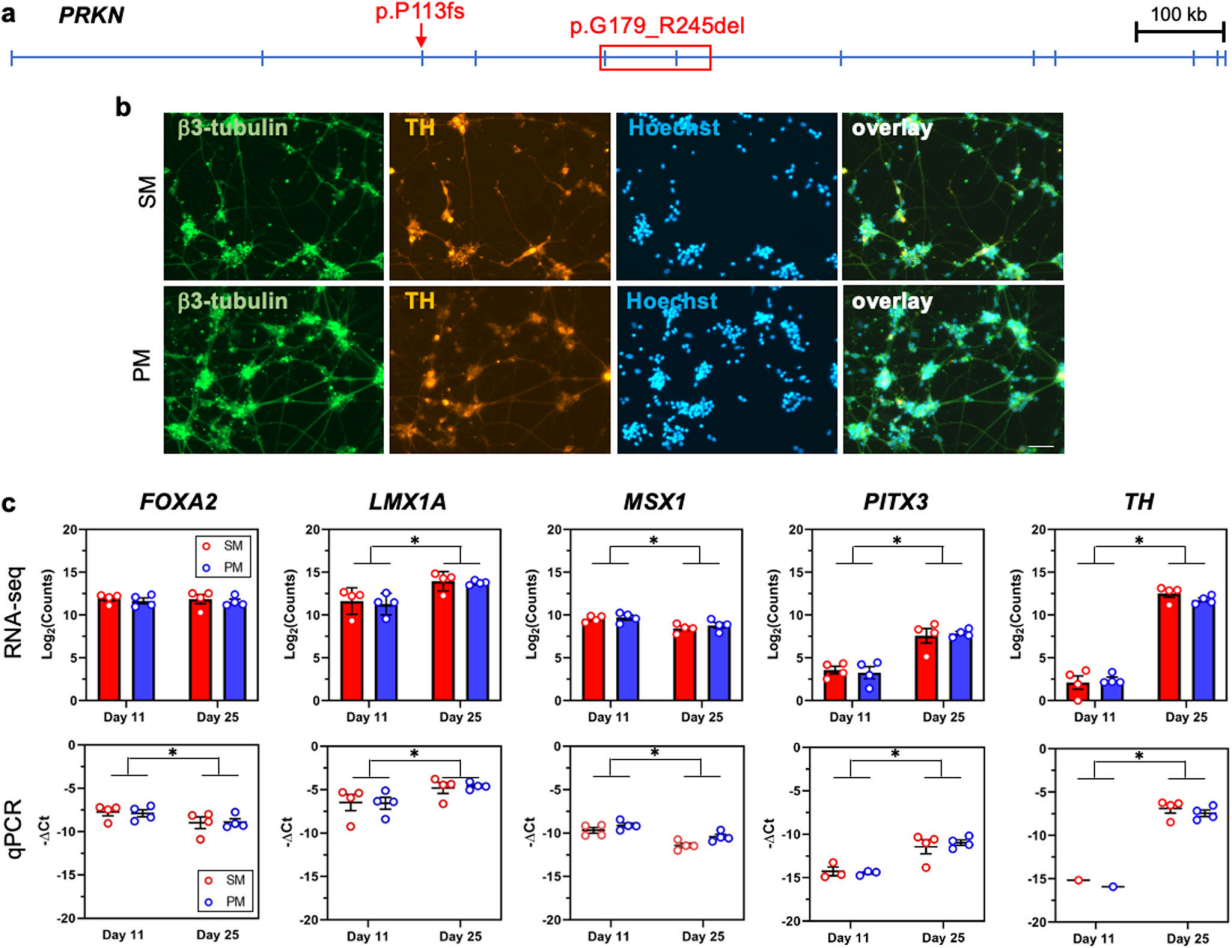
Differentiation of SM and PM hiPSC into dopamine neurons. **a** *PRKN* gene, transcript variant 1 that encodes a 465 amino acid protein, is shown with the compound heterozygous mutations identified in the brothers: p.P113fs in exon 3 and p.G179_R245del that spans exons 5 and 6. **b** The expression of β3-tubulin (green) and tyrosine hydroxylase (TH, red) of dopamine neurons differentiated for 27–28 days was assessed by immunocytochemistry. All cultures were counterstained with the nuclear Hoechst stain (light blue). Shown here are cell lines SM15 and PM18, scale bar = 50 µm. **c** mRNA of dopamine neuron lineage markers expressed by day 11 and day 25 SM (red) and PM (blue) neuronal cultures derived from four SM- and PM hiPSC lines each were quantified by RNA-seq and RT-qPCR. RNA-seq counts were multiplied by a factor of 100 to accommodate logarithmic scaling on the y-axis of the graphs. On day 11 1/4 (PITX3) and 3/4 (TH) of the samples for SM and PM were below the threshold of RT-qPCR detection and are thus not depicted on the graphs. Except for *FOXA2* (RNA-seq), the expression levels for all dopamine neuron markers were statistically significantly different between day 11 and day 25, but were not significantly different between SM and PM for RT-qPCR and RNA-seq. Two-way ANOVA followed by Sidak’s multiple comparisons *post-hoc* test (mean ± SEM; p* < 0.05).

We have previously reported on two brothers (PM and SM) who carry identical compound heterozygous mutations in *PRKN*, a 40 base pair deletion in exon 3 (rs771529549, p.P113fs) and a deletion encompassing exons 5 and 6 (p.G179_R245del), both predicted to result in deleterious frameshift mutations^20,36^. Here, the precise deletion in exon 3 was confirmed by wholeexome sequencing (WES, Fig. 1a, Table 1). Moreover, the larger deletion in exon 5 and 6 was refined through copy number variant (CNV) analysis. While the precise breakpoints were not established, the minimum size of the deletion encompassing exons 5 and 6 is over 100,000 base pairs (Fig. 1a, Table 1). hiPSC lines were derived from each brother and all lines were validated through karyotyping and by Pluritest (Supplementary Fig. 1)^37^. Additional RT-qPCR and immunocytochemical analysis demonstrating the expression of pluripotency markers were previously reported^36,38^.

**Table 1:** *PRKN* Deletion Coordinates in PD Brothers.

individual	deletion position (Hg38)	size (base pairs)	location in *PRKN* gene	protein consequence	method
PM	chr6:162,262,561–162,262,600	40	exon3:c.337_376del	p.P113fs	WES
	chr6:161,950,810–162,054,690	103,880	exon5–6:c.535_734del	p.G179_R245del	CNV analysis
SM	chr6:162,262,561–162,262,600	40	exon3:c.337_376del	p.P113fs	WES
	chr6:161,927,578–162,054,690	127,112	exon5–6:c.535_734del	p.G179_R245del	CNV analysis

### Differentiation of SM and PM hiPSCs into mesencephalic dopamine neurons

hiPSCs were differentiated into mesencephalic dopamine neurons in a two-step process that included a midbrain patterning resulting in day 11 mesencephalic neural precursors (floor plate cells) followed by further differentiation into day 25 early postmitotic mesencephalic dopamine neurons^39,40^. The differentiations, as assessed by immunocytochemistry were of high efficacy (Fig. 1b, Supplementary Fig. 2)^39^. Quantification of the expression of additional mesencephalic dopaminergic neuronal markers by RT-qPCR (Fig. 1c), shows decreasing levels for *FOXA2* and *MSX1*, and increasing expression for *LMX1A*, *PITX3* and *TH* between day 11 and day 25 of differentiation, as previously reported by us and others^39,40^. The expression of all five markers at both time points were not significantly different between SM and PM lines, suggesting equal efficiency of differentiation to the early mesencephalic lineage for SM and PM hiPSC lines. The data from the RT-qPCR and RNA-seq analysis of dopamine neuron developmental markers correlated significantly (Figs. 1c, 5c).

### Genomic variants in genes of interest

Whole exome sequencing (WES) identified variants at 29,732 genomic positions; 20,599 variants were present in both, SM and PM and the remaining 9,133 positions were unique to one of the brothers (PM: 4,271 and SM: 4862, Fig. 2, Supplementary Table 1). Thirty-four of the variants were located within 14 of the established PD-relevant genes (Table 2)^3^. Of those 34 variants, 21 were identical in SM and PM, including the previously reported c.337_376del frameshift deletion in *PRKN* (rs771529549). Eight variants were unique to PM and three to SM, all of which were reported to be either benign or tolerated by the ClinVar database, SIFT program or PolyPhen-2 program.

**Fig. 2:**
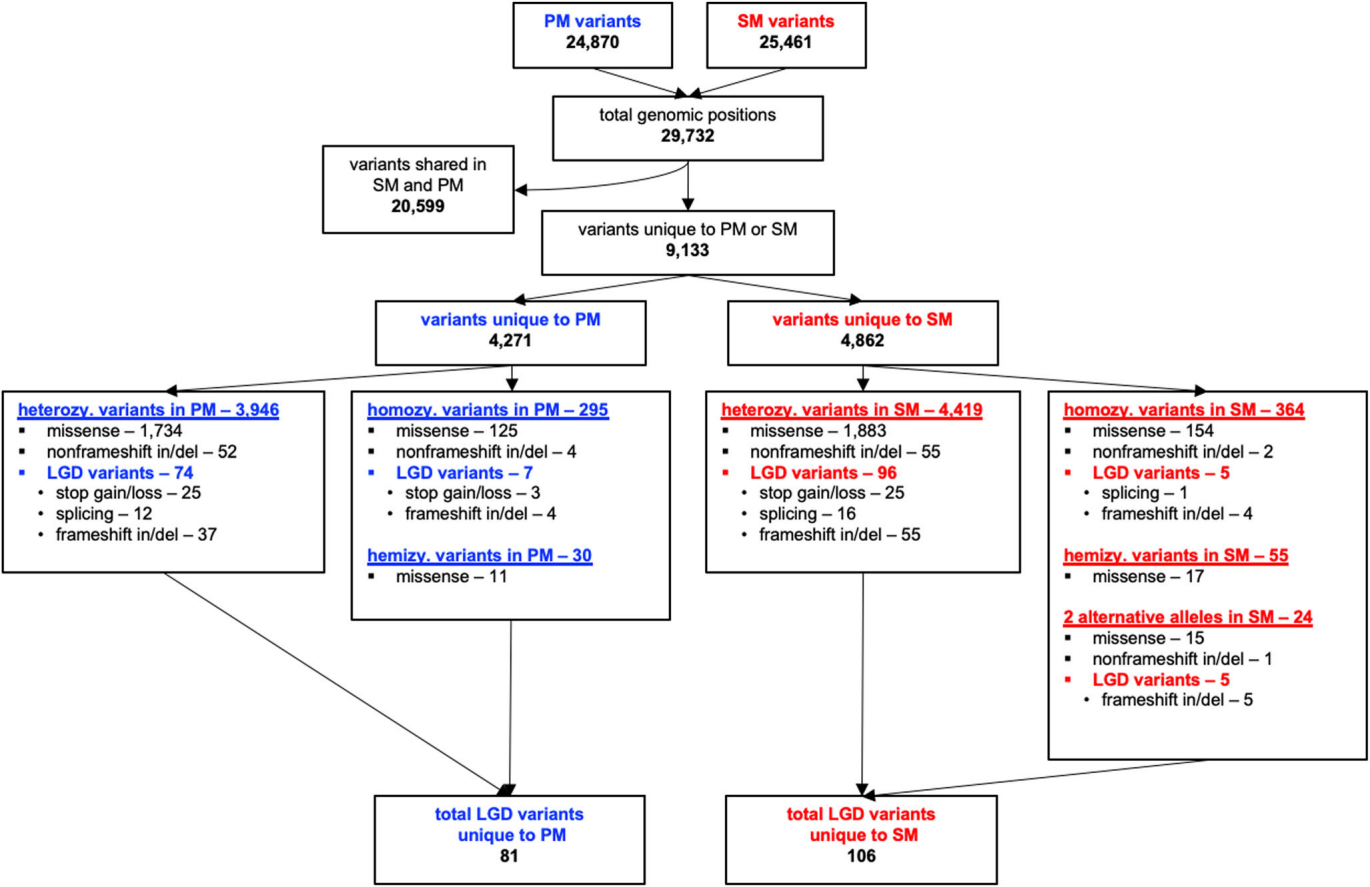
Whole exome sequencing data prioritization overview. All variants detected for each of the siblings were compared and those unique to SM or PM further evaluated with regards to the type of mutation. The total number of variants that were likely gene disrupting (LGD) for SM (106) and PM (81) are indicated. (heterozy heterozygous, homozy homozygous, hemizy hemizygous).

**Table 2:** Variants Identified in Known PD Genes by WES.

carrier of variant	gene	position (Ch37/hg19)	nucleotide change	amino acid change	function	SNP	PM genotype ^(1)^	SM genotype ^(1)^	gnomAD allele frequency	ClinVar	SIFT	Polyphen-2	CADD
only in PM	*ATP13A2*	chr1:17314702-17314702	c.G2790A	p.S930S	synonymous	rs3738815	0/1	0/0	0.000008	Benign	–	–	–
	*DNAJC6*	chr1:65858145-65858145	c.G1290A	p.E430E	synonymous	rs4325172	0/1	0/0	0.453500	–	–	–	–
	*EIF4G1*	chr3:184039666-184039666	c.A1315G	p.M439V	missense	rs2178403	0/1	0/0	0.758700	Benign	Tolerated	Benign	0.001
	*FBXO7*	chr22:32871365-32871365	c.G17A	p.G6E	missense	rs9621461	0/1	0/0	0.000033	–	Tolerated	Benign	8.137
	*FBXO7*	chr22:32875190-32875190	c.G345A	p.M115I	missense	rs11107	0/1	0/0	0.000008	Benign	Tolerated	Benign	0.422
	*FBXO7*	chr22:32887150-32887150	c.C949T	p.L317L	synonymous	rs9726	0/1	0/0	0.447800	Benign	–	–	–
	*LRRK2*	chr12:40688695-40688695	c.T2857C	p.L953L	synonymous	rs7966550	0/1	0/0	0.102900	Benign	–	–	–
	*PINK1*	chr1:20960230-20960230	c.C189T	p.L63L	synonymous	rs45530340	0/1	0/0	0.171800	Benign	–	–	–
only in SM	*DNAJC6*	chr1:65867519-65867519	c.G1973A	p.S658N	missense	rs4915691	0/0	0/1	0.173300	–	Tolerated	Benign	4.757
	*LRRK2*	chr12:40631791-40631791	c.T457C	p.L153L	synonymous	rs10878245	0/0	0/1	0.520100	Benign	–	–	–
	*PINK1*	chr1:20977000-20977000	c.A1562C	p.N521T	missense	rs1043424	0/0	0/1	0.291700	Benign	Tolerated	Benign	14.4
identical in PM and SM	*DNAJC13*	chr3:132218623-132218623	c.G4387T	p.A1463S	missense	rs3762672	1/1	1/1	0.565000	–	Tolerated	Benign	14.37
	*DNAJC6*	chr1:65860687-65860687	c.A1800C	p.P600P	synonymous	rs4582839	0/1	0/1	0.647200	–	–	–	–
	*EIF4G1*	chr3:184037533-184037533	c.A502G	p.T168A	missense	rs13319149	1/1	1/1	0.998000	–	Tolerated	Benign	1.882
	*GIGYF2*	chr2:233659553-233659553	c.C1378A	p.P460T	missense	rs2289912	0/1	0/1	0.052480	–	Tolerated	Benign	15.79
	*GIGYF2*	chr2:233708806-233708806	c.A2940G	p.Q980Q	synonymous	rs3816334	0/1	0/1	0.000018	–	–	–	–
	*GIGYF2*	chr2:233712227-233712229	c.3630_3632del	p.1210_1211del	nonframeshift deletion	rs10555297	0/1	0/1	–	Benign	–	–	–
	*LRRK2*	chr12:40629436-40629436	c.T356C	p.L119P	missense	rs33995463	0/1	0/1	0.001256	Benign	Deleterious	Possibly damaging	27.5
	*LRRK2*	chr12:40713834-40713834	c.C4872A	p.G1624G	synonymous	rs1427263	0/1	0/1	0.682600	Benign	–	–	–
	*LRRK2*	chr12:40757330-40757330	c.A7155G	p.G2385G	synonymous	rs33962975	0/1	0/1	0.113600	Benign	–	–	–
	*LRRK2*	chr12:40758652-40758652	c.T7190C	p.M2397T	missense	rs3761863	0/1	0/1	0.619100	Benign	Tolerated	Benign	0.005
	*LRRK2*	chr12:40619082-40619082	c.G149A	p.R50H	missense	rs2256408	1/1	1/1	0.990400	Benign	Tolerated	Benign	13.86
	*PLA2G6*	chr22:38508249-38508249	c.C2340T	p.N780N	synonymous	rs138683183	0/1	0/1	0.007564	Benign	–	–	–
	***PRKN***	**chr6:162683593-162683632**	**c.337_376del**	**p.P113fs**	**frameshift deletion**	**rs771529549**	**0/1**	**0/1**	**0.000124**	**Pathogenic**	–	–	–
	*VPS13C*	chr15:62207911-62207911	c.T8366C	p.I2789T	missense	rs72747885	0/1	0/1	0.000018	Benign	Tolerated	Benign	2.797
	*VPS13C*	chr15:62212781-62212781	c.A7128G	p.Q2376Q	synonymous	rs17238189	0/1	0/1	0.000004	–	–	–	–
	*VPS13C*	chr15:62254989-62254989	c.A3394G	p.I1132V	missense	rs3784635	0/1	0/1	0.070070	–	Tolerated	Benign	5.977
	*VPS13C*	chr15:62259637-62259637	c.G2921A	p.R974K	missense	rs3784634	0/1	0/1	0.617800	–	Tolerated	Benign	7.541
	*VPS13C*	chr15:62299603-62299603	c.A1194C	p.I398I	synonymous	rs9635356	0/1	0/1	0.072590	–	–	–	–
	*VPS13C*	chr15:62316035-62316035	c.G458A	p.R153H	missense	rs12595158	0/1	0/1	0.072090	–	Damaging	Possibly damaging	33
	*VPS13C*	chr15:62202482-62202482	c.G8738A	p.S2913N	missense	rs10851704	1/1	1/1	0.559800	–	Tolerated	Benign	12.1
	*VPS35*	chr16:46696284-46696284	c.C1938T	p.H646H	synonymous	rs168745	1/1	1/1	0.992100	Benign	–	–	–
heterozygous in SM and homozygous in PM	*SYNJ1*	chr21:34003928-34003928	c.4215_4216insAATACT	p.L1406delinsNTL	nonframeshift insertion	rs71640263	1/1	0/1	0.000008	–	–	–	–
	*UCHL1*	chr4:41259633-41259633	c.C53A	p.S18Y	missense	rs5030732	1/1	0/1	0.228900	Benign	Tolerated	Benign	20.8

^(1)^ Homozygous wild type genotypes are denoted with 0/0, heterozygous varaint genotypes are denoted as 0/1, and homozygous variant genotypes are denoted as 1/1. *PRKN* deletion highlighted in bold.

In addition to variants in established PD-relevant genes, other unique variants being “likely gene-disrupting” (LGD) and predicted to result in stop-gain (nonsense), stop-loss, frameshift, or splicing alterations are highlighted as variants that might have the most significant effect on protein function. PM has 81 unique LGD variants and SM has 106 LGD variants (Fig. 2, Supplementary Table 2). Across both brothers, 39 of the 187 total LGD alterations are within the highly polymorphic *HLA* genes. Of note, we identified a splicing alteration (rs74853476) in the *dopamine β* (*DBH*) gene in PM. This rare variant replaces a nucleotide two base pairs downstream of the first exon of *DBH*, has a CADD score of 23, and is predicted to be damaging by the MutationTaster program.

### Comparison of gene expression of SM and PM dopamine neurons on day 11 and day 25 of differentiation

**Fig. 3:**
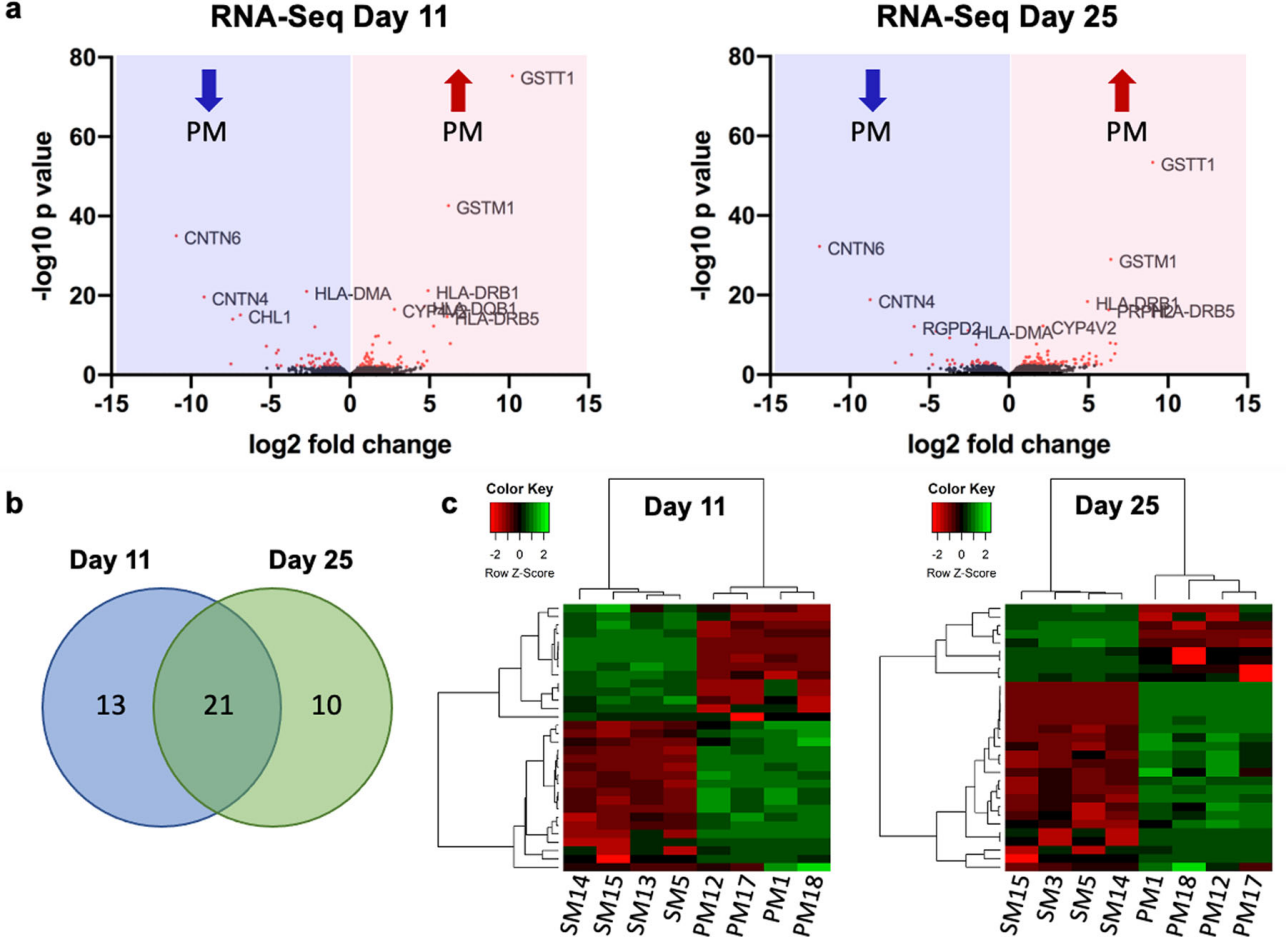
RNA-seq at days 11 and 25 of dopaminergic differentiation. **a** Volcano plots illustrate the total number of genes sequenced (black) and genes showing significantly increased or decreased (red) expression levels in PM versus SM (*p* < 0.01) on day 11 and day 25 of differentiation. The top 10 significant genes are identified on the plots. **b** Venn diagram indicating that 21 genes are differentially expressed in PM and SM on both days of differentiation, while 13 and 10 genes are differentially expressed on days 11 and 25, respectively (FDR < 0.05) **c** Heat map of the significantly differentially expressed genes (FDR < 0.05).

Levels of gene expression were compared between PM and SM cultures on days 11 and 25 of dopamine neuron differentiation (Fig. 3, Tables 3 and 4, Supplementary Fig. 3). 34 and 31 genes were significantly differentially expressed on days 11 and 25, respectively (FDR < 0.05). 21 of those were differentially expressed at both time points in the same direction, with 13 genes being upregulated and 8 genes downregulated in PM compared to SM. 13 and 10 genes were differentially expressed only on day 11 and day 25, respectively (Fig. 3b, Tables 3 and 4). A heatmap of the differentially expressed genes shows concordance among hiPSC lines derived from the same patient (Fig. 3c).

**Table 3:** Genes significantly differentially expressed at Day 11.

	gene ^(1)^	fold change	logCPM	FDR
**higher expression in PM**	***GSTT1***	**1181.555**	**2.859**	**3.05E-71**
	***FAM203B***	**79.294**	**−2.136**	**2.79E-05**
	***GSTM1***	**72.933**	**1.901**	**4.75E-39**
	***HLA-DRB5***	**69.095**	**0.031**	**8.61E-12**
	***PRPH2***	**38.286**	**−0.733**	**1.71E-09**
	***HLA-DRB1***	**29.902**	**1.426**	**6.74E-18**
	***HLA-DQB1***	**25.687**	**1.801**	**3.65E-14**
	*EN2*	24.833	−1.268	0.002406
	*ZIC1*	9.049	4.494	0.024157
	***CYP4V2***	**6.957**	**2.168**	**1.66E-13**
	***AL592284.1***	**5.643**	**−0.294**	**1.65E-05**
	***ECHDC3***	**3.412**	**2.897**	**4.10E-07**
	***CRYZ***	**3.048**	**5.666**	**5.08E-07**
	***TYW3***	**2.674**	**5.345**	**4.45E-05**
	***TAF9B***	**2.608**	**4.866**	**0.003647**
	*HLA-B*	2.265	5.024	0.037139
	*SMN2*	2.109	2.732	0.014611
	*CDC6*	2.091	5.060	0.004974
	*IFITM3*	1.977	6.455	0.020864
**higher expression in SM**	*NBPF14*	−2.163	3.855	0.00921
	*ULK4*	−2.262	2.715	0.003733
	*ADAMTS13*	−2.267	2.334	0.009936
	*BEX5*	−3.002	0.722	0.005235
	***HIST1H4C***	**−4.637**	**2.504**	**2.49E-09**
	*PRSS45*	−5.253	−1.057	0.013989
	*FTCD*	−6.067	0.382	0.011038
	***HLA-DMA***	**−6.625**	**2.391**	**8.70E-18**
	***GBP3***	**−22.815**	**−0.880**	**0.000979**
	*GRM7*	−24.666	−2.066	0.004436
	***RGPD2***	**−38.065**	**0.410**	**0.0001**
	***CHL1***	**−118.195**	**3.107**	**3.22E-12**
	***NPIPB15***	**−165.246**	**−1.242**	**4.04E-11**
	***CNTN4***	**−577.971**	**0.373**	**1.65E-16**
	***CNTN6***	**−1942.680**	**3.667**	**1.49E-31**

^(1)^ genes highlighted in bold were significantly different at both time points.

**Table 4:** Genes significantly differentially expressed at Day 25.

	gene ^(1)^	fold change	logCPM	FDR
**higher expression in PM**	***GSTT1***	**526.895**	**2.336**	**2.47E-49**
	***HLA-DRB5***	**334.958**	**−0.532**	**1.96E-13**
	*MS4A6E*	105.167	−1.925	3.37E-05
	*HOXA5*	101.040	−2.021	0.006575
	***GSTM1***	**84.507**	**1.473**	**1.68E-25**
	***FAM203B***	**82.813**	**−2.200**	**2.26E-05**
	***PRPH2***	**77.661**	**−0.331**	**2.49E-13**
	*PSCA*	44.304	−2.803	0.02524
	*TBX1*	33.243	0.830	0.005335
	***HLA-DRB1***	**30.871**	**1.288**	**2.63E-15**
	*CYP4F31P*	25.413	−1.756	0.028118
	*SLC18A2*	5.501	3.984	0.001665
	*TTPA*	5.399	−1.043	0.010922
	***HLA-DQB1***	**5.340**	**0.879**	**0.002105**
	***CYP4V2***	**4.395**	**3.748**	**2.09E-09**
	*NOS2*	3.807	3.434	0.04549
	***AL592284.1***	**3.588**	**0.155**	**0.006579**
	*PCDHB8*	3.459	2.251	0.033791
	***CRYZ***	**3.301**	**5.311**	**3.49E-05**
	***TYW3***	**2.447**	**4.071**	**0.031666**
	***ECHDC3***	**2.311**	**2.699**	**0.040708**
	***TAF9B***	**2.109**	**5.284**	**0.033791**
**higher expression in SM**	***HIST1H4C***	**−4.150**	**1.703**	**4.71E-05**
	***HLA-DMA***	**−5.890**	**1.601**	**2.74E-08**
	***GBP3***	**−13.262**	**-0.637**	**1.31E-06**
	***NPIPB15***	**−23.796**	**−0.974**	**4.02E-08**
	*TMEM257*	−29.055	−2.050	0.010644
	***RGPD2***	**−62.154**	**0.733**	**2.96E-09**
	***CHL1***	**−70.111**	**6.960**	**0.010644**
	***CNTN4***	**−424.412**	**5.744**	**1.04E-15**
	***CNTN6***	**−3837.681**	**2.895**	**1.15E-28**

^(1)^ genes highlighted in bold were significantly different at both time points.

*Glutathione S-transferase theta 1* (*GSTT1*) was the most- and *glutathione S-transferase mu 1* (*GSTM1*) was among the most significantly upregulated genes in PM relative to SM cultures on day 11 and day 25, while *contactin 4* (*CNTN4*), *contactin 6* (*CNTN6*) and neural cell adhesion molecule L1-like protein (*CHL1*) were the most significantly down-regulated genes in PM at both time points (Fig. 3a, Tables 3 and 4).

**Fig. 4:**
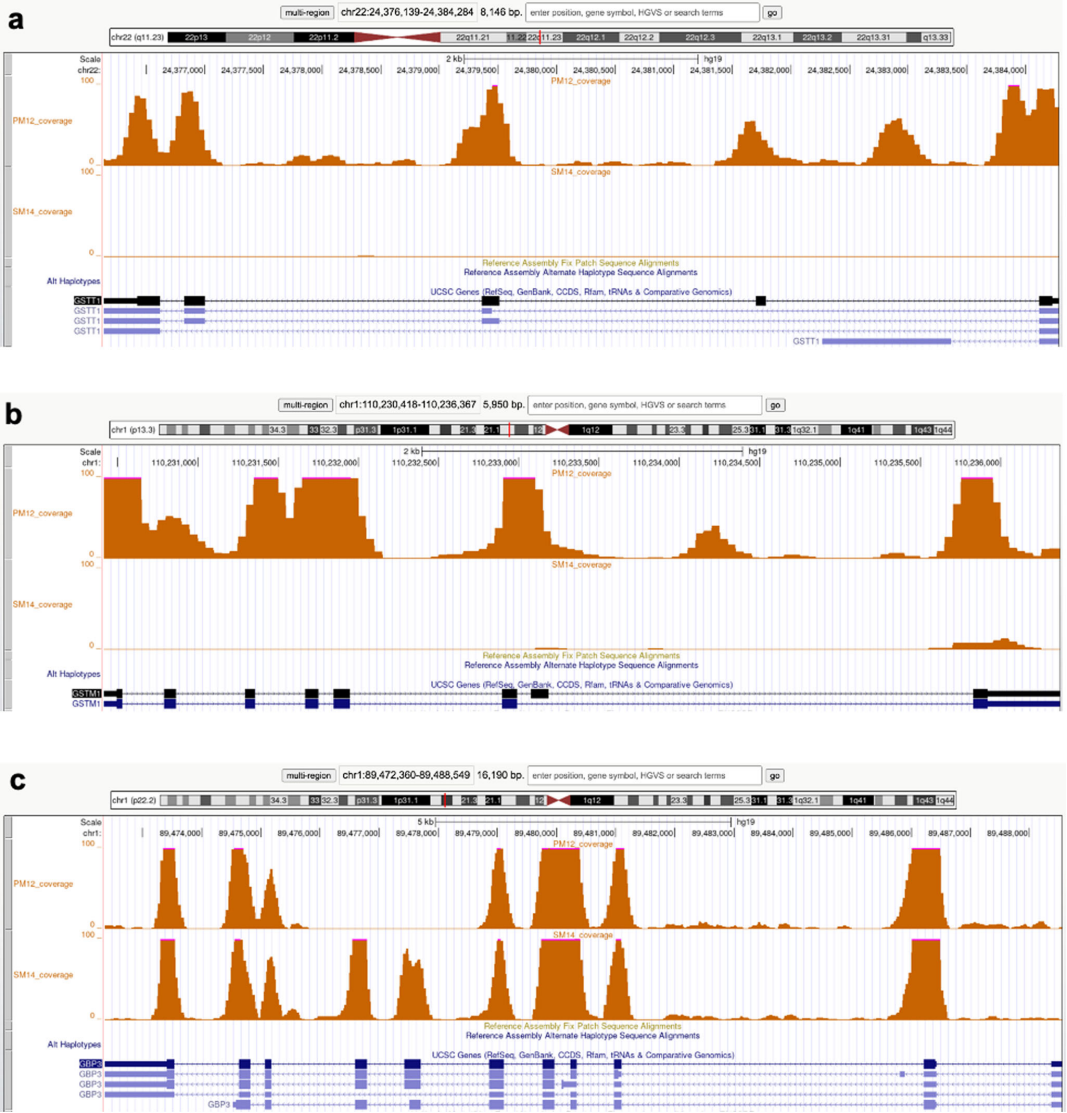
Large copy number variants. **a**, **b** UCSC genome browser (Hg38) visualizing the coverage for the hiPSC lines PM12 and SM14 from whole exome sequencing with orange peaks that align with the locations of exons. SM is missing both copies of the *GSTT1* (**a**) and *GSTM1* **b** genes. **c** PM is missing coverage across exons 7 and 8 in *GBP3*.

Each significantly differentially expressed gene identified by RNA-seq was evaluated for potential genomic changes that may be driving its expression levels. Examination of the WES coverage data revealed that SM was homozygous null across the entire length of the *GSTT1* and *GSTM1* genes, which likely explains the large deficit of expression in SM when compared to PM (Fig. 4a and b). PM has a homozygous deletion encompassing exons 7 and 8 in the *guanylate binding protein 3* (*GBP3*) gene, predicted to result in a frameshift across all transcripts of this gene (Fig. 4c). *GBP3* was expressed at significantly lower levels in PM on both, day 11 and day 25 (Tables 3 and 4).

When we overlaid the differentially expressed genes (FDR < 0.05) with the variants from the wholeexome sequencing, 282 variants were identified as being unique to one of the brothers (Supplementary Table 3). For *GBP3*, we found, in addition to the above-mentioned deletion encompassing exons 7 and 8, three nonsynonymous variants, two of which had CADD scores >10 p.R225W (SM, 0/1) and p.R221Q (SM, 0/1). *CHL1*, one of the cell adhesion genes significantly downregulated in PM (Tables 3 and 4) showed one nonsynonymous variation in PM but with a very low CADD score (0.003, Supplementary Table 3). The other most significantly down-regulated genes in PM, *CNTN4* and *CNTN6,* showed several different variants. Three of the four *CNTN4* variants were distributed among the two brothers (p.P190P, SM 0/1; pN294N, PM1/1, SM 0/1; p.R402R PM 0/1) and had CADD scores between 11–12, while both *CNTN6* variants had very low CADD scores (Supplementary Table 3).

The vast majority of variants (224/282) fall in the highly polymorphic *HLA* genes. *HLA-DRB5* was the most differentially expressed *HLA* gene, with PM showing 69-fold and 335-fold higher expression than SM on days 11 and 25, respectively. We detected a total of 48 different variants in *HLA-DRB5*, with 16 showing CADD scores > 10; of those, 15 were present in SM and only 1 in PM (Supplementary Table 3). For the other differentially expressed *HLA* genes, we made the following observations: HLA-B: 9/49 variants have CADD scores >10 and 8/9 of those were present in SM; *HLA-DMA:* 3/3 variants have CADD scores >10 and 3/3 were present in PM; *HLA-DQB1*: 11/54 variants have CADD scores >10 and of those 10/11 were present in SM; *HLA-DRB1*: 10/70 variants had CADD scores >10 and 10/10 of those were present in SM. Overall, for all five of those *HLA* genes, the individual carrying the majority of variants showed lower expression of the respective *HLA* gene (Tables 3 and 4 and Supplementary Table 3). In summary, of the 25 genes identified to have brother-specific variants, 19 and 17 genes showed significantly different expression levels between the brothers on day 11 and 25, respectively; of the 16 genes with variants with CADD scores > 10, twelve and eleven genes showed significantly different expression levels between SM and PM on days 11 and 25, respectively.

**Fig. 5:**
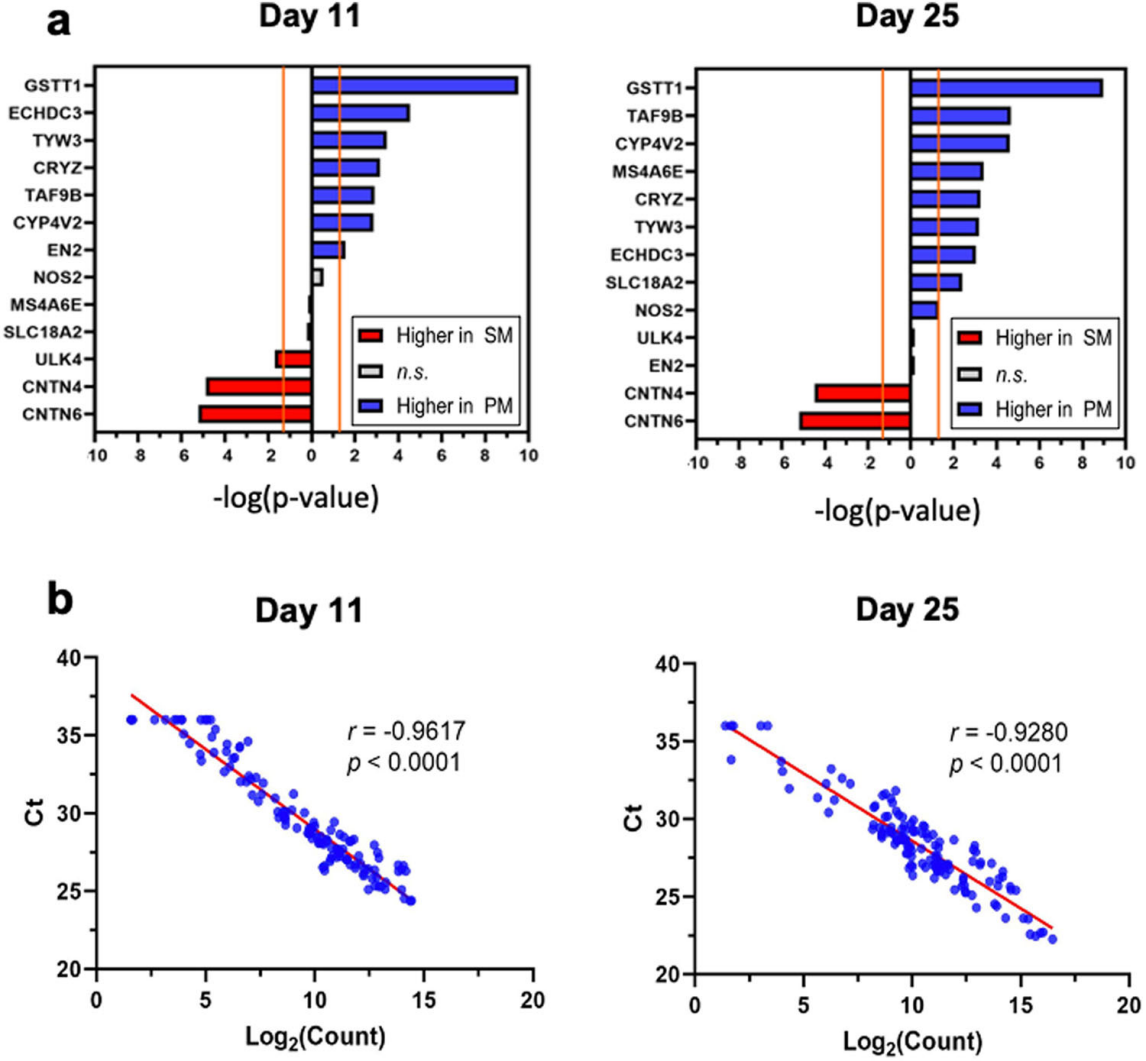
RT-qPCR validation of RNA-seq findings. **a** 13 of the genes identified as differentially expressed in SM and PM by RNA-seq on day 11 and day 25 of differentiation were validated by RT-qPCR. The statistical significance (-log *p* values) of the differential expression between PM and SM cultures evaluated are graphed. Grey-colored bars lying within the range of non-significant differences as delineated by two orange lines were not significantly differently expressed in PM and SM cultures as assessed by Rt-qPCR (*p* > 0.05). **b** Correlation analysis of the expression levels of all genes analyzed by RT-qPCR and RNA-seq including the dopamine-lineage markers (Fig. 1c) across all four SM and PM hiPSC lines are graphed for day 11 and day 25 (Log_2_(Count) versus Ct; Pearson correlation analysis, two-tailed *p*-value). RNA-seq counts were multiplied by a factor of 100 to accommodate logarithmic scaling on the x-axis of the graphs (**c**).

Using a combination of three in silico predictive programs (SIFT, Polyphen2, MutationTaster) to predict damaging or pathogenic variants, we identified three nonsynonymous variants which had a CADD score greater than 10: *GBP3* p.R225W (SM) and *ULK4* p.S348G (SM), and *MS4A6E* p.V47F (heterozygous in PM and homozygous in SM). *GBP3* was expressed at significantly higher levels in SM on both day 11 and 25, while *ULK4* was expressed significantly higher in SM on day 11 only and *MS4A6E* was higher in PM on day 25 only (Fig. 5a, b, Tables 3 and 4). Thus, while all three of these alterations are fairly common, with minor allele frequencies in ExAC of 0.12 or higher, they may still potentially be acting as modifiers.

We selected a subset of 13 genes found to be differentially expressed by RNA-seq between PM and SM either on both days (8 genes) or day 11 (2 genes, *EN2*, *ULK4*) or day 25 only (3 genes, *NOS2, MS4A6E*, *SLC18A2*) and validated the differential expression for all 13 genes by RT-qPCR (Fig. 5a). The RT-qPCR data for all the genes evaluated, including the 5 lineage markers (Fig. 1c) showed a strong correlation with the RNA-seq data (Fig. 5b).

### Metabolomics

**Fig. 6:**
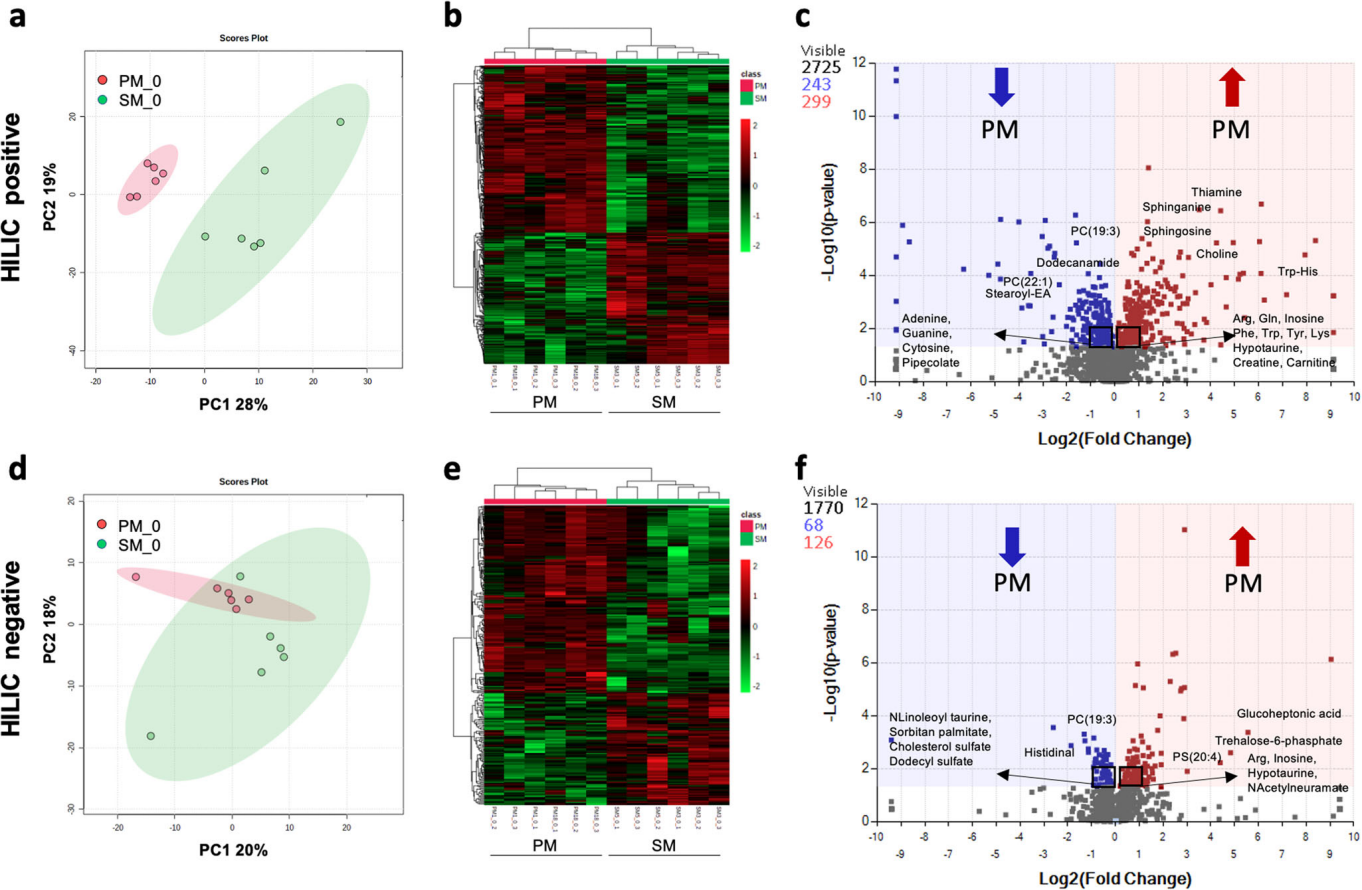
The metabolome of SM and PM neural precursors show significant differences. The metabolome of SM and PM day 11 neural precursors resulting from three separate differentiations (1–3) were analyzed by HILIC positive mode (**a**–**c**) and HILIC negative mode (**d**–**f**) mass-spectrometry. **a**. **d** Principal component analysis (PCA plots) illustrates distinct metabolomic profiles for PM vs SM. **b**, **e** Hierarchical clustering maps provide global comparisons for PM vs SM differences of the top 500 significantly different individual metabolite levels (rows) clustered by SM and PM cell lines and differentiations (columns). Metabolites are colored according to relative feature abundance across all samples ranging from low (green) to high (red). **c**, **f** Volcano plots illustrate the total number of detected metabolites (gray) and significantly increased (red) or decreased (blue) metabolites in PM vs SM (*p* < 0.05). A total of 2725 (HILIC pos) and 1,770 (HILIC neg) compounds were detected of which 542 (HILIC pos) and 194 (HILIC neg) are significantly different between PM vs SM. Representative members of the identified pathways (Fig. 7) altered between the two brothers are highlighted. Some of the most significantly changed molecules cannot not be annotated, because there are too many isomers as possible candidate metabolites.

Our RNA-seq data demonstrates that already on day 11 of differentiation there are important differences in gene expression between SM and PM lines. In order to assess effects downstream of the transcriptional differences in very early stages of dopaminergic differentiation, we performed metabolomics of day 11 cultures. Day 11 cultures are more homogenous than their day 25 counterparts and thus provide a greater opportunity to detect differences in metabolites. Thus, we performed two independent metabolomics analyses of day 11 neural precursor cells (floor plate cells) using two different types of liquid chromatography separation and two different sets of SM and PM hiPSC lines. These analyses revealed significantly different metabolomic profiles between the two brothers (Fig. 6a, d). The relative abundance of metabolites was concordant within different hiPSC lines derived from the same individual (Fig. 6b, e). A similar number of metabolites were elevated or decreased in PM when compared to SM and representative metabolites belonging to pathways altered between the two brothers (see below) are highlighted in the volcano plots (Fig. 6c, f**;** Supplementary Fig. 4a, b). In particular we observed 4.2 and 1.4-fold lower levels of GSH and GSSG, respectively for PM vs SM, which translates into a 3-fold lower GSH/GSSG ratio for PM as compared to SM floor plate cells (Supplementary Table 4). We further measured 1.6-fold higher level of glutamine in PM than SM cells, similar to observations made in in vivo NMR analysis of the putamen metabolome in PD patients^41^. We also detected increased levels of creatine in PM, such measurements in PD patients are contradictory^42,43^.

**Fig. 7:**
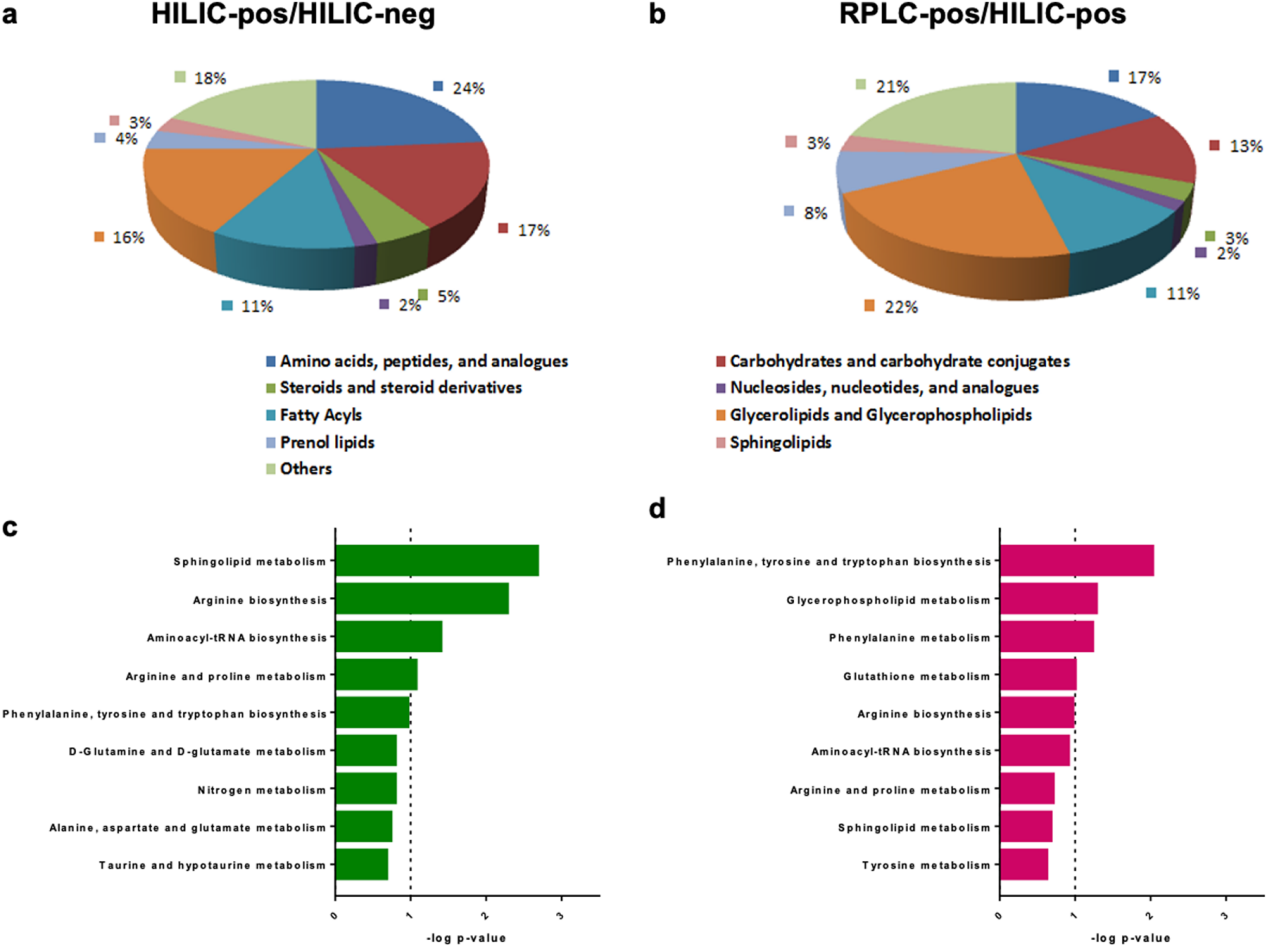
Biochemical classification and pathway analysis of metabolites differentially regulated in SM and PM neural precursors. **a**, **b** Metabolites significantly different between PM and SM were classified according to their biochemical properties and **c**, **d** pathway analysis was performed. The top 9 significant response pathways are shown for each data set with a total of 8 pathways being significantly different between SM and PM (*p* ≤ 0.1 is indicated by the dashed line).

Metabolites present at statistically significantly different levels (*p* < 0.05) between PM and SM developing dopamine neuron cultures were classified according to their biochemical properties. For the hydrophilic interaction liquid chromatography positive/negative ion mode (HILIC pos/neg) analysis (method #2), the largest percentage of metabolites belonged to the lipid and lipid-like molecules (34%), with the second-largest group (24%) identifying as amino acids, peptides and analogues (Fig. 7a). The same overall distribution was observed for the reverse-phase liquid chromatography (RPLC) pos/HILIC pos analysis (method #1) although, not surprisingly, the lipid and lipid-like molecules were present more prominently (44%), and the amino acids, peptides and analogues contributed to a lesser degree (17%) (Fig. 7b).

### Pathway analysis

**Fig. 8:**
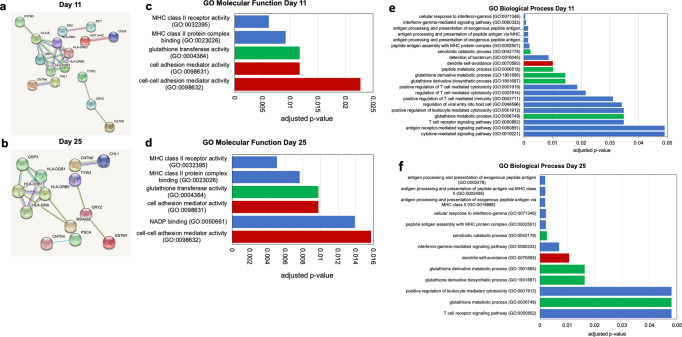
Pathway analysis of significantly differentially expressed genes. **a**, **b** STRING networks with differentially expressed genes on day 11 (**a**) and day 25 (**b**). Only those genes connected to a node are shown. **c**, **d** Statistically significantly different GO Molecular Functions and **e**, **f** GO Biological Processes (FDR < 0.05) determined from the differentially expressed genes for day 11 (**c**, **e**) and day 25 (**d**, **f**) of differentiation are plotted. Pathways relating to GSH metabolism are indicated in green, neural development in red and all others in blue. The GO Biological Processes labelled in blue involve MHC class II gene expression, genes which have been demonstrated to play a role in neural development independent of their function in cell immunity.

Pathway analysis was performed separately for the metabolomic and RNA-Seq data (Figs. 7, 8). The metabolite pathways identified to be different between the two brothers were qualitatively similar between the two chromatography methods (method #1 and #2) but ranked slightly differently. Thus, sphingolipid metabolism was identified as the most significantly different pathway for the HILIC pos/neg analysis (method #2), but did not reach statistical significance for the RPLC pos/HILIC pos analysis (method #1) (Fig. 7c, d). The biosynthesis of aromatic amino acids (phenylalanine, tyrosine and tryptophan) was identified as the most significantly different pathways by RPLC pos/ HILIC pos, but did not reach statistical significance in the HILIC pos/HILIC neg analysis (Fig. 7c, d). RPLC pos/HILIC pos analysis identified glutathione metabolism as significantly different between SM and PM (Fig. 7d). GSH metabolism was also identified in the RNA-seq based GO analysis for Biological Processes and Molecular Functions for day 11 and day 25 cultures (Fig. 8a–f). GO analysis of the RNA-seq data further identified cell adhesion and dendrite self-avoidance, processes which play important roles in neuronal development (Fig. 8a–f). In addition, several pathways and processes relating to immune function were identified that were driven by several *HLA* genes (Fig. 8a–f). The *HLA* gene products most likely play a role in neural development independent of their function in cellular immunity (see discussion).

## Discussion

The remarkable difference in the clinical presentation with respect to age of onset and severity of symptoms between the brothers SM and PM who carry the same compound heterozygous *PRKN* mutations and have lived in the same geographical area for the majority of their lives, led to the hypothesis of the presence of additional genetic elements that could potentially play a role in the differential onset and progression of PD in these two patients.

There is increasing evidence that there is a developmental component to PD pathogenesis^21–25^. Proper brain development requires finely tuned interactions among hundreds of genes and a dysregulation in these networks can lead to neurodevelopmental disorders^44^. Indeed, many PD-associated genes have been implicated in neuronal developmental processes^45,46^. We identified a number of differentially expressed genes between SM and PM developing dopamine neurons that are either directly implicated in dopamine neuron development, neural development in general, or are candidate genes for neurodevelopmental functions.

The most disparate gene expression between SM and PM developing dopamine neurons was observed for a group of cell adhesion proteins *CNTN6*, *CNTN4* and *CHL1*, that were expressed at levels orders of magnitude lower in PM than SM neurons at both stages of differentiation (day 11 and day 25) suggesting persistent differential expression during development (Tables 3 and 4). Intriguingly, *CHL1* was previously shown to be differentially regulated in the substantia nigra of brains from PD patients compared to control brains^47^. Another study using living tissue from frontal lobe biopsies of patients and controls demonstrated a significant difference in the expression of *CNTN6*^48^. Thus, two of these three genes have been independently verified to be differentially expressed in PD patients. *CNTN6*, *CNTN4* and *CHL1* encode neuronal membrane immunoglobulin-like proteins that play a role in axon guidance and outgrowth, orientation of dendrites and synaptogenesis in the developing nervous system^49–52^ and are expressed in the developing human embryonic substantia nigra pars compacta (https://www.brainspan.org)^51–53^. Mesencephalic dopamine neurons extend their axons from the substantia nigra into the dorsal striatum where they form extensive ramified axonal branches^54,55^. Thus, a defect in axon pathfinding or synapse formation could potentially have a significant impact on mesencephalic-striatal circuit formation and connectivity. Variants of each of these three cell adhesion molecules in isolation have been implicated in several different neurodevelopmental disorders^49,53,56^. We observed a concomitant and rather large difference in the expression of all three of these genes between SM and PM dopamine neuron cultures, an observation that supports our hypothesis of a potential difference in the development of the dopaminergic system between the two brothers.

Differential expression *of the gene EN2* in the prefrontal cortex of patients with PD compared to controls has been reported^57^ and the analysis of human haplotype implicates variations in the engrailed 2 gene *(EN2*) in the development for early-onset PD^58^. The development of mesencephalic dopamine neurons is tightly orchestrated by the spatiotemporal expression of highly conserved transcription factors such as engrailed 1 and 2, which are involved in early lineage specification and axon guidance^23,59–63^. We observed *EN2* expression levels in day 11, but not day 25 cells to be 20-fold higher in PM than SM cells. Dysregulation of *EN1/2* expression in either direction has been shown to interfere with the development of mesencephalic dopamine neurons^61,64–66^. However, since the differential *EN2* expression was only observed on day 11 but not day 25 of differentiation, we cannot exclude the possibility that this higher expression reflects a slightly different time frame of development between SM and PM lines, rather than a significant overall developmental difference.

Human *HLA* genes are ubiquitously expressed and their developmentally regulated expression throughout the brain including the ventral mesencephalon suggests an immune system unrelated role for these proteins in early neuronal differentiation^67–83^. Our observation that several of the HLA genes were differentially expressed between in SM and PM (Table 4) so early in dopamine neuron differentiation raises the possibility of potential differences in midbrain development between PM and SM. Enhanced expression of HLA genes has been shown to lead to aberrations in neurodevelopment^74^. In this context, it is interesting to point out that the majority of the *HLA* genes differentially expressed on day 11 (4 out of 5) and on day 25 (3 out of 4) were expressed at higher levels in PM than SM cultures. Importantly, three human *HLA* II genes (*HLA-DQB1, HLA-DRB1*, and *HLA-DRB5*) that we observed to be differentially expressed are located within the chromosome 6p21.3 major histocompatibility complex class II cluster and were previously reported to be a top GWAS hit for late-onset PD^84–89^. Moreover, evidence points to the possibility of multiple PD risk-associated variants in this region including both coding and noncoding, regulatory alterations^89,90^.

We identified variants at a total of 29,732 positions, and of those, 34 were located on 14 established PD-relevant genes several of which carried multiple variants (Table 2). We identified 8 variants that were unique to PM and 3 for SM. Although the large majority of these 34 variants on their own are classified as benign or tolerated, it is not clear how the combination of multiple variants within one gene affects protein levels and/or function. The significance of the presence of multiple variants in these PD-relevant genes for the clinical discordance between PM and SM is difficult to deduce and would require protein expression- and functional studies.

Very striking was the differential expression of *GSTM1* and *GSTT1*, which we demonstrate to be the result of SM being homozygous null for both genes; thus, although the RNA samples analyzed represent a snapshot during early nigral development, the fact that the lack of expression of these two genes are due to null mutations implies that these two enzymes are permanently lacking in SM. Both genes encode glutathione S-transferases, enzymes involved in the protection against xenobiotics and oxidative stress, cellular processes that have been correlated with PD pathogenesis^91–96^. These large-scale deletions are relatively common in white, non-Hispanic populations (~50% for *GSTM1* and ~20% for *GSTT1*)^97^ but studies on their role in PD pathogenesis are conflicting^98–102^.

Another interesting variant representing a potential modifier of the discordant clinical presentation is *DBH*, a gene that encodes the enzyme dopamine beta-hydroxylase (DBH) that converts dopamine to norepinephrine. In this study, a rare splicing variant (rs74853476) was identified in PM that may disrupt the splice donor site adjacent to exon 1. Risk assessments of *DBH* variants for PD are conflicting^103–110^. To our knowledge the *DBH* variant (rs74853476) we observed in PM has not been assessed for its PD risk potential.

Our metabolomics pathway analysis identified three biochemical processes differentially regulated between SM and PM: 1) sphingolipid and glycerophospholipid metabolism, 2) the metabolism of several amino acids including phenylalanine, tyrosine, tryptophan, arginine and proline together with aminoacyl-tRNA biosynthesis and 3) GSH metabolism which was also identified in the RNA-seq-based GO analysis. A decreased GSH/GSSG ratio is an indicator of oxidative stress, a mechanism believed to play a role in PD pathogenesis. We observed a 3-fold lower GSH/GSSG ratio for PM as compared to SM floor plate cells. Very similar observations have been reported for day 25 *PRKN-KO* hiPSC-derived mesencephalic dopamine neurons^111^, as well as for PD patient tissue^112^. The absence of functional parkin causes abnormal mitochondrial morphology and function resulting in increased oxidative stress^36,113^. Thus, an additional depletion of GSH, the most abundant antioxidant in the CNS, could therefore add additional stress on dopaminergic neurons of patient PM versus SM. Importantly in the context of this study, aberrant GSH metabolism has not only been associated with PD^91,96,102^ but also serves as an indicator of developmental toxicity^114^. The metabolism of aromatic amino acids has been shown to be different between PD patients and control subjects in most metabolite profiling studies, the directionality of these differences however, are not always concordant^43^. Alterations in mitochondrial aminoacyl-tRNA biosynthesis resulting from mutations in mitochondrial aminoacyl-tRNA synthetases cause a variety of pathogenic CNS phenotypes including infantile-onset parkinsonism^115–117^. In addition, a protein complex interacting with aminoacyl-tRNA synthetase, a substrate of parkin, accumulates in parkin-inactivated PD animal models and causes dopamine neuron degeneration^118,119^. The metabolism of lipids, including glycerophospholipids and sphingolipids, has been reported to be altered in PD patients^120,121^ and importantly, has been found to play a central role in all mechanisms presently believed to underly PD pathogenesis^121–127^. Sphingolipids and glycerophospholipids are major constituents of the plasma membrane and not surprisingly, are important for neural maintenance and development, playing roles in cell-cell recognition, cell adhesion, signal transduction and synapse formation^122,128–133^.

The authors acknowledge that the differences in gene variants, gene transcription and metabolism between SM and PM reported here are not functionally validated in this current study and, thus, do not provide causal evidence for the clinical divergence between the brothers. However, our observations contribute to a basis of PD associated genetic, transcriptional and metabolomic phenotypes that builds the foundation for the establishment of causal relationships between genetic and biochemical cellular phenotypes and clinical observations. Indeed, our and other groups’ reports of PD patient dopamine neurons genetic and biochemical phenotypes which have also been described in PD patients suggests that these human in vitro approaches can serve as models to establish causal relationships between genetic differences and PD pathogenesis.

In summary, we assessed genetic differences that could potentially play a role in the drastically different clinical presentations of two brothers carrying the same compound heterozygous *PRKN* mutations. Combining exon sequencing, transcriptomics and metabolomics we found differences in gene expression (*CNTN4, CNTN6*, *CHL1*, *HLA* genes) and metabolism (sphingo- and glycerophospholipids) relevant to the development of mesencephalic dopamine neurons. We further observed differences in cellular processes underlying dopamine homeostasis (*DBH*, tyrosine biosynthesis) and protection against insults from oxidative stress and xenobiotics (*GSTM1* and *GSTT1*, GSH metabolism), cellular functions that have been shown to play a role in PD pathogenesis. Future studies should be aimed at examining cellular consequences of these genetic differences on the development, function and vulnerability of these human dopamine neurons.

## Methods

### Patient Information

We have previously published a detailed description of the patients PM and SM^20^. In brief, PM and SM are brothers, aged 6 years apart who share the same compound heterozygous disease-causing mutations in *PRKN*^20^. PM displayed significant postural and gait impairment in his mid 20 s and was diagnosed with PD in his mid 30 s. At age 42 he started with bilateral subthalamic nucleus deep brain stimulation and at age 50 obtained additional bilateral deep brain stimulation in the globus pallidus interna. Since age 53 the patient is functionally wheelchair-bound. His six-year younger brother SM developed exercise-induced foot dystonia which was resolved by selegiline. Examined at age 47 he did not meet UK Brain Bank Criteria for PD^35^.

### Derivation, validation and differentiation of hiPSCs

hiPSC lines were derived from dermal fibroblasts from two human subjects, PM and SM, who underwent a dermal biopsy at age 46 and 40, respectively, after providing the appropriate patient consent/assent under the guidelines of an approved Internal Review Board (IRB) protocol at Vanderbilt University (#080369). Four independent hiPSC clones from each patient were used in this study (PM1, PM12, PM17, PM18 and SM3, SM5, SM14 and SM15). Three of the hiPSC lines (PM1, SM3, SM5) were generated by transducing the fibroblasts with a lentivirus as previously described^38^. The other five lines (PM12, PM17, PM18, SM14, SM15) were reprogrammed by electroporation with CXLE plasmid vectors (Addgene) using the Neon Transfection System (Life Technologies) following published methods^39,134^. Karyotype analyses were performed for all hiPSC lines using standard protocols with at least 20 metaphase spreads per cell line (Genetics Associates). The lack of plasmid integration into the genomic DNA was demonstrated by RT-qPCR. The pluripotency of all hiPSC lines was validated by Pluritest^37^ and/or by analyzing the expression of pluripotency markers by immunofluorescence and RT-qPCR. In addition, the capacity of the hiPSC lines to differentiate into cell types belonging to the three germ layers was assessed^36,38,135^.

Differentiation of the hiPSCs to a mesencephalic dopaminergic lineage was performed as previously described^39,40,136^. In brief, hiPSCs were differentiated in a first stage into floor plate cells (mesencephalic neural precursors) (days 0–11) via dual SMAD inhibition combined with ventral midbrain patterning. In a second stage, these floor plate cells were further differentiated (days 11–25) into early postmitotic mesencephalic dopamine neurons. The RNA-seq data were derived from two independent dopamine neuron differentiations that were performed with two different sets of SM and PM hiPSC lines each; thus, we analyzed a total of four SM and four PM lines (Supplementary Fig. 3). The metabolomics data which corroborate some of the RNA-seq data were obtained from an additional two independent differentiations. Thus, the data presented in this manuscript is based on four independent dopamine neuron differentiations.

### Immunofluorescence

For immunofluorescence analysis, dopamine neurons were plated into 96-well plates (Greiner Bio-One) and immunofluorescence was performed as previously described^39^. Briefly, the cells were fixed in PBS containing 4% paraformaldehyde (Electron Microscopy Sciences) for 30 min at room temperature, permeabilized with 0.2% Triton X-100 for 20 min at room temperature and then incubated in PBS containing 5% normal donkey serum (Jackson ImmunoResearch) and 0.05% Triton X-100 for 2 h at room temperature or overnight at 4 °C. The following primary antibodies were used: mouse anti-β3-tubulin (Thermo Scientific; MA1-19187, 1:500), rabbit anti-tyrosine hydroxylase (TH) (Pel-Freez; P40101, 1:500) and sheep anti-TH (Pel-Freez; P60101, 1:250). Secondary antibodies conjugated to DyLight 488 (1:800), and DyLight 549 (1:800), both from Jackson ImmunoResearch. Images were obtained with a Zeiss ObserverZ1 microscope and AxioVs40 software (version 4.7.2). For high content imaging images were acquired using a Molecular Device’s ImageXpress Micro XL system and MetaXpress software available at the Vanderbilt Highthroughput Screening Core Facility. Quantification of β3-tubulin- and TH-positive cells was performed in two cultures for each cell line and at least 9000 cells per culture.

### DNA extraction and wholeexome sequencing

Whole exome sequencing (WES) was performed on the PM12 and SM14 lines. Briefly, 3 μg of DNA was extracted from hiPSCs using the DNeasy Blood & Tissue Kit (Qiagen) and exonic DNA regions were selected using the Agilent SureSelect Human All Exon 60 Mb v6 kit according to the manufacturer’s instructions. The DNA was sequenced on the Illumina HiSeq 2500 with 100 base pair (bp) paired-end reads. Data were processed according to Genome Analysis Tool-kit (GATK) best practices with variants required to be called in both brothers, to have a GQ score of 99 or above, and were annotated using SeattleSeq^137^. All variants were compared to a list of 21 established PD genes^3^. Variants were then prioritized for those that were unique to either brother, “likely gene-disrupting” (LGD), and predicted to result in stop-gain (nonsense), stop-loss, frameshift, or splicing mutations.

### Copy number variant (CNV) analysis

DNA isolated from the PM12 and SM14 clones for WES was also used for genotyping with the Illumina 1M-Single array. Samples had average call rates >98%. CNVs were identified using the cnvPartition v.3.2.0 algorithm implemented in Illumina GenomeStudio software. Select genes and regions of interest were also manually evaluated using the pile up data generated from the whole exome sequencing. Manual visualization of WES coverage was also used to identify large, homozygous deletions within a subset of genes that were found to be significantly different in the transcriptomic analysis.

### RNA extraction and transcriptomic analysis

Total RNA was extracted from day 11 floor plate cells and day 25 early post-mitotic dopaminergic neurons differentiated from all eight hiPSC lines using the Qiagen RNeasy Mini Kit according to the manufacturer’s protocol. Cell lysates were homogenized using QIAshredder spin columns (Qiagen) and genomic DNA was removed by an on-column DNase treatment (Qiagen). The concentration and quality of RNA samples was assessed using a Bioanalyzer RNA kit (Agilent Technologies) and RNA integrity numbers for all RNA samples ranged between 8.3 to 10. cDNA libraries were generated with the Illumina TruSeq Stranded kit with a poly-A selection. Paired-end 100 base pair read sequencing was performed on indexed samples and run on the HiSeq 2500, yielding a minimum of 25 million reads/sample. Alignment of RNA-seq reads against the human genome (GRCh38) was performed using STAR Aligner and Htseq-count was used to count the number of overlapping reads with genes. Differential expression analysis was conducted using edgeR with a false discovery rate (FDR) cut-off of 0.05 to correct for the multiple comparison testing^138^.

### Quantitative RT-PCR

RT-qPCR and transcriptomic analyses were performed on the same RNA samples. 3 µg of total RNA were used for each sample for reverse transcription (RT) into cDNA using iScript Reverse Transcription Supermix for RT-qPCR (Bio-Rad) according to the manufacturer’s instructions. RT-qPCR reactions were performed in triplicates at 1.5 ng/µl cDNA using SsoAdvanced Universal SYBR Green Supermix (Bio-Rad) at a final volume of 10 µl on the Bio-Rad CFX384 Touch™ Real-Time System in 384-well standard PCR plates. Melting curves for each reaction were visually inspected via Bio-Rad CFX Maestro™ 1.1 Software and the acceptable range of Ct values for each RT-qPCR triplicates was implemented assuming 100% primer efficiency as described^139^. Relative quantification of genes of interest was conducted using actin as a reference gene and fold change was calculated using the 2^-∆∆Ct method, setting the arithmetic mean ∆Ct value of SM/PM as calibrator. Collectively, for genes with RT-qPCR triplicates showing no amplification signal or a mean Ct value of ≥ 36, a mean Ct value of 36 was imputed for statistical analyses as previously described^139^. Two-tailed Student’s t-tests were performed to determine statistical significance for each gene with *p* value < 0.05 as the cut-off for significance and not assuming consistent standard deviation between groups. For the analysis of dopaminergic lineage markers, ordinary two-way ANOVA followed by Sidak’s multiple comparison *post-hoc* test was conducted using ∆Ct values. In all cases, data were presented as mean ± SEM. Correlation analyses between RNA-seq and RT-qPCR results were performed via Pearson’s correlation analysis.

### Metabolomics

#### Cell harvest and metabolite extraction

Optima grade LC-MS solvents for the mass spectrometry analyses were obtained from Thermo Fisher Scientific (Fair Lawn, NJ). Two separate metabolomics analyses using slightly different methods were performed with different SM and PM hiPSC lines.

For method #1 (RPLC positive ion mode /HILIC positive ion mode) day 11 neural dopaminergic precursor cells (floor plate cells of one well of a 6 well plate) were harvested into 500 µl of ice-cold methanol, flash frozen and then stored at −80 °C. To extract the metabolites, the 500 µl methanol cell suspensions were thawed and 100 µl of H_2_O added. Then the samples were frozen on dry ice for 3 min, defrosted in ice over a 10 min period, and sonicated with 10 pulses using a probe sonicator at 30% power. The freeze-thaw-sonication sequence was repeated three times. The proteins were precipitated by placing the lysates at −80 °C overnight and then pelleted by centrifugation at 25,000 x g for 15 min. Cleared supernatants containing the metabolites were placed in clean Eppendorf tubes, dried in a vacuum concentrator and stored frozen at −80 °C. For RPLC positive ion mode mass spectrometry analysis the dried extracts were reconstituted in 60 μl of RPLC buffer (acetonitrile/water with 0.1% formic acid, 2:98, v/v). Samples were vortexed rigorously to solubilize the metabolites, cleared by centrifugation for 5 min at 15,000 rpm, and the supernatants were injected twice (5 μl / injection) randomly. Quality control samples were prepared by combining equal volumes (10 μl) of each sample. After RPLC mass-spectrometry, the remaining samples were dried down *in vacuo*, and the metabolites reconstituted in 40 μl of HILIC buffer (acetonitrile/water, 90:10, v/v) and 5 µl of each sample was injected twice in random sequence to perform HILIC positive ion mode mass-spectrometry.

In method #2 (HILIC positive ion mode/negative ion mode) the cells were washed three times with 2.5 ml of an ammonium formate buffer (50 mM, pH 6.8), scraped into the same buffer, centrifugated at 200 x for 5 min, the cell pellets flash frozen in liquid nitrogen and stored at −80 °C. To extract the metabolites, cell pellets were lysed in 200 µl ice-cold lysis buffer (1:1:2, Acetonitrile:MeOH:Ammonium Bicarbonate 0.1 M, pH 8.0, LC-MS grade) and sonicated once as described above. The protein concentration was determined (BCA assay, Thermo Fisher Scientific) and adjusted to 1 mg/ml. Isotopically labeled standard molecules, Phenylalanine-D8 and Biotin-D2 were added to the 200 µl cell lysates, the protein precipitated by addition of 800 µl of ice-cold methanol and stored at −80 °C overnight. Upon thawing, the precipitated proteins were pelleted by centrifugation at 9300 x g for 10 min, the supernatants transferred into two clean Eppendorf tubes, dried down *in vacuo* and stored at −80 °C. To perform HILIC-positive ion mode and HILIC-negative ion mode mass spectrometry, each sample was reconstituted in 60 μl of HILIC reconstitution buffer (acetonitrile/water, 90:10, v/v) and 5 µl of each sample was injected once for positive ion mode and 8 µl for negative ion mode. During the final reconstitution, isotopically labeled standard molecules, Tryptophan-D3, Carnitine-D9, Valine-D8, and Inosine-4N15, were added to each sample, and quality control sample was prepared by pooling equal volumes from each individual sample.

#### Mass spectrometry and data acquisition

UPLC-IM-MS and data-independent acquisition (MS^E^) were performed on a Waters Synapt G2 HDMS (Milford, MA, USA) mass spectrometer equipped with a Waters nanoACQUITY UPLC system and autosampler (Milford, MA, USA). Metabolites were separated on a reverse phase 1 mm × 100 mm HSS T3 C18 column packed with 1.8-μm particles (Waters, Milford, MA, USA) held at 45 °C. Liquid chromatography was performed using a 30-min gradient at a flow rate of 75 μl min^−1^ using solvent A (0.1% formic acid in H_2_O) and solvent B (0.1% formic acid in acetonitrile). A 1 min wash period (99% solvent A) was performed prior to any gradient changes. After 1 min, solvent B increased to 60% over 10 min and up to 99% over another 10 min. The column was re-equilibrated to 99% solvent A for 5 min after each run. IM-MS^E^ analyses were run in resolution mode, with a capillary voltage of 2.75 kV, source temperature at 100 °C, sample cone voltage at 30 V, extraction cone voltage at 5 V, source gas flow of 400 ml min^−1^, desolvation gas temperature of 325 °C, He cell flow of 180 ml min^−1^, and an IM gas flow of 90 ml min^−1^. The data were acquired in positive ion mode from 50 to 2000 Da with a 1 s scan time; Leucine enkephalin was used as the lock mass (*m/z* 556.2771 in ES + mode) at a concentration of 2 ng ml^−1^ infused at a flow rate of 7 μl min^−1^. All analytes were analyzed using MS^E^ with an energy ramp from 10 to 40 eV.

High resolution (HR) MS and data-dependent acquisition analyses were performed on a high-resolution Q-Exactive HF hybrid quadrupole-Orbitrap mass spectrometer (Thermo Fisher Scientific, Bremen, Germany) equipped with a Vanquish UHPLC binary system and autosampler (Thermo Fisher Scientific, Germany).

For HILIC analysis both positive and negative ion mode, metabolite extracts were separated on a SeQuant ZIC-HILIC 3.5-μm, 2.1 mm × 100 mm column (Millipore Corporation, Darmstadt, Germany) held at 40 °C. Liquid chromatography was performed at a 200 μl min^−1^ using solvent A (5 mM Ammonium formate in 90% water, 10% acetonitrile) and solvent B (5 mM Ammonium formate in 90% acetonitrile, 10% water) with the following gradient: 95% B for 2 min, 95–40% B over 16 min, 40% B held 2 min, and 40–95% B over 15 min, 95% B held 10 min (gradient length 45 min).

Full MS analyses were acquired over a mass range of m/z 70–1050 using electrospray ionization both positive and negative ion mode. Full mass scan was used at a resolution of 120,000 with a scan rate of 3.5 Hz. The automatic gain control (AGC) target was set at 1 × 10^6 ^ions, and maximum ion injection time was at 100 ms. Source ionization parameters were optimized with the spray voltage at 3.0 kV, and other parameters were as follows: transfer temperature at 280 °C; S-Lens RF level at 40; heater temperature at 325 °C; Sheath gas at 40, Aux gas at 10, and sweep gas flow at 1. Tandem mass spectra were acquired using a data-dependent scanning mode in which one full MS scan (m/z 70–1050) was followed by 2, 4 or 6 MS/MS scans. MS/MS scans were acquired in profile mode using an isolation width of 1.3 *m/z*, stepped collision energy (NCE 20, 40), and a dynamic exclusion of 4 s. MS/MS spectra were collected at a resolution of 15,000, with an automatic gain control (AGC) target set at 2 × 10^5 ^ions, and maximum ion injection time of 100 ms. The retention times and peak areas of the isotopically labeled standards were used to assess data quality.

#### Metabolite data processing and analysis

The acquired UPLC-IM-MS^E^ raw data and LC-HR MS/MS raw data were imported, processed, normalized and reviewed using Progenesis QI v.2.1 (Nonlinear Dynamics, Newcastle, UK). All MS and MS/MS sample runs for one particular analysis (RPLC or HILIC, positive or negative ion mode) were aligned against a quality control (pooled) reference run, and peak picking was performed on individual aligned runs to create an aggregate data set. Unique ions (retention time and m/z pairs) were grouped using both de-adduction and de-isotoping to generate unique “features” (retention time and m/z pairs) representative of unannotated metabolites. Data were normalized to all features using Progenesis QI. Compounds with <30% coefficient of variance (%CV) were retained for further analysis *P* values were calculated by Progenesis QI using variance stabilized measurements achieved through log normalization, and metabolites with a *p*-value ≤ 0.1 (method #1) and *p* value ≤ 0.05 (method #2) calculated by a one-way analysis of variance (ANOVA) test were considered significant. Metabolomic data has been filtered to remove known mass spectrometry contaminants^140^.

Tentative and putative annotations were determined within Progenesis QI software using accurate mass measurements (<5 ppm error), isotope distribution similarity, and fragmentation spectrum matching database searches against Human Metabolome Database (HMDB)^141^, METLIN^142^ the National Institute of Standards and Technology (NIST) database^143^ and an in-house library. Annotations from both RPLC and HILIC analyses were performed for all significant compounds (*p* value ≤ 0.1 or ≤ 0.05). Annotated metabolites were further analyzed by pathway overrepresentation analysis using MetaboAnalyst 4.0^144^. In these experiments, the level system for metabolite identification confidence was utilized. Briefly, many annotations were considered to be tentative (level 3, L3) when a top candidate cannot be prioritized^145^, but they still represent families of molecules representative for the data acquired. The annotations considered putative (level 2, L2) and validated (level 1, L1) are for molecules with a fragmentation spectrum matching one of the databases or a standard molecule from the in-house library. All metabolite measurements and their annotations are uploaded (Supplementary Table 4).

### Pathway analysis

Significantly differentially expressed genes were evaluated in the STRING v11 database, a program that correlates the direct and indirect associations found between proteins^146^. GO 2021 pathways were determined using the publicly available Enrichr database^147,148^.

## Supplementary information

Supplementary Figures

Cukier et al., 2022 Data set 1

Cukier et al., 2022, Data set 2

Cukier et al., 2022 Data set 3

Cukier et al., 2022 Data set 4

## Data Availability

Raw RNA sequencing data and processed gene counts that support the findings of this study are available through the Gene Expression Omnibus at GSE184694. Metabolomics data are available at the NIH Common Fund’s National Metabolomics Data Repository (NMDR) Web site, the Metabolomics Workbench, https://www.metabolomicsworkbench.org where it has been assigned Study ID (ST001957).
