# Supplementary material for: Genomic, transcriptomic, and metabolomic profiles of hiPSC-derived dopamine neurons from clinically discordant brothers with identical PRKN deletions
Source: NPJ Parkinsons Dis. 2022 Jun 29;8:84. doi: 10.1038/s41531-022-00346-3 (PMC9243035; doi:10.1038/s41531-022-00346-3)
Supplement: Supplementary file 1 — Supplementary Figures [file 41531_2022_346_MOESM1_ESM.pdf]

Supplementary Figure 1

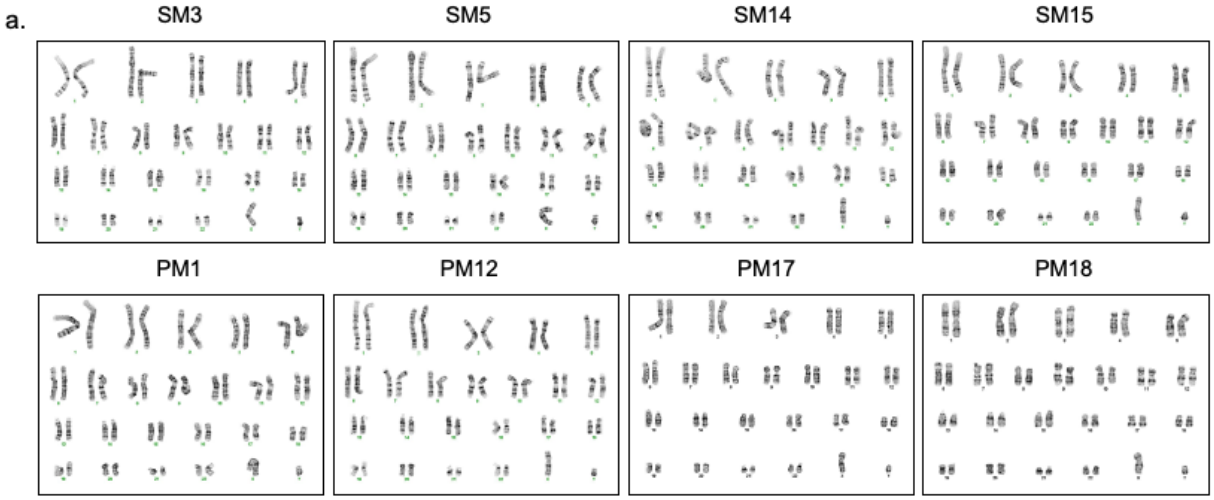

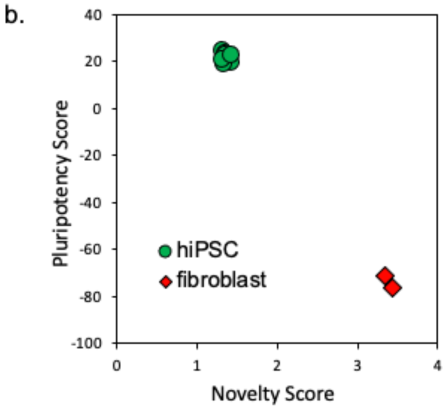

c.

Cell line	Novelty Score	Pluripotency Score
SM3	1.31	24.61
SM14	1.35	23.57
SM15	1.42	19.83
PM1	1.35	22.83
PM11	1.33	18.67
PM12	1.32	20.91
PM17	1.43	22.97
CA fibroblast	3.43	-76.16
CA fibroblast	3.35	-71.06

Supplementary Figure 1. Validation of hiPSC lines. All four SM- and PM hiPSC lines have a normal karyotype (**a**). The pluripotency of seven of the hiPSC lines was assessed by the Pluritest (**b**) and all the hiPSC lines show Pluripotency- and Novelty Scores within the expected range, whereas fibroblast scores are, as expected, outside the required range (**c**).

Supplementary Figure 2

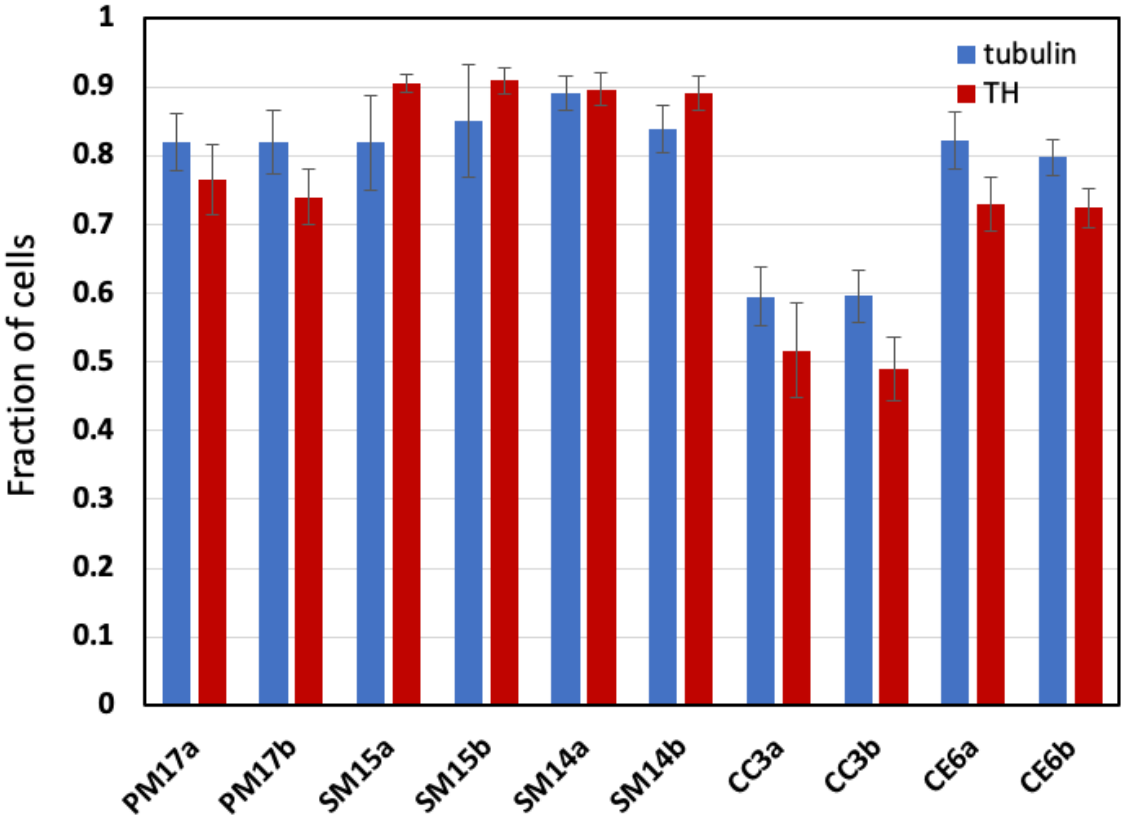

Supplementary Figure 2. Efficacy of differentiation of SM and PM hiPSC lines into mesencephalic dopaminergic neurons. The number of β3-tubulin (blue) and tyrosine hydroxylase (TH, red)-positive cells was quantified by high content imaging in an independent differentiation. Quantification of β3-tubulin and TH-positive cells was performed in two cultures (a and b) for each cell line and 16 images for each culture, and a total of > 9000 cells per culture were analyzed (shown are means ± STDEV). The efficiencies of the differentiation of the *PARK2* lines (SM and PM lines) was higher than the ones observed for co-differentiated, co-stained and co-imaged control lines (CC3, CE6).

Supplementary Figure 3

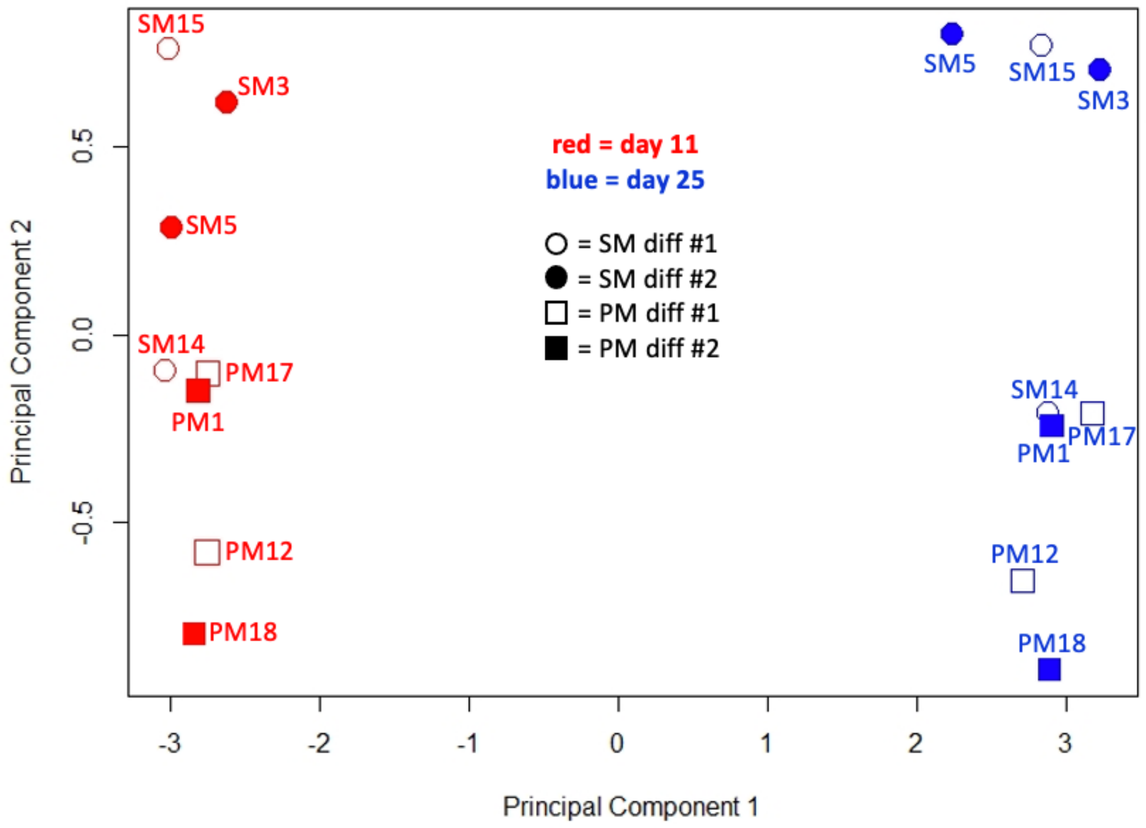

Supplementary Figure 3. Principal component analysis (PCA) of gene expression data. The PCA plot illustrates distinct expression profiles for day 11 (red) and day 25 (blue) of differentiation (PCA1) and between SM and PM lines (PCA2). There is no separation between data points from the two separate differentiations performed (empty and filled symbols) or hiPSC lines derived using a viral (single digit lines) or episomal (double digit lines) method.

Supplementary Figure 4

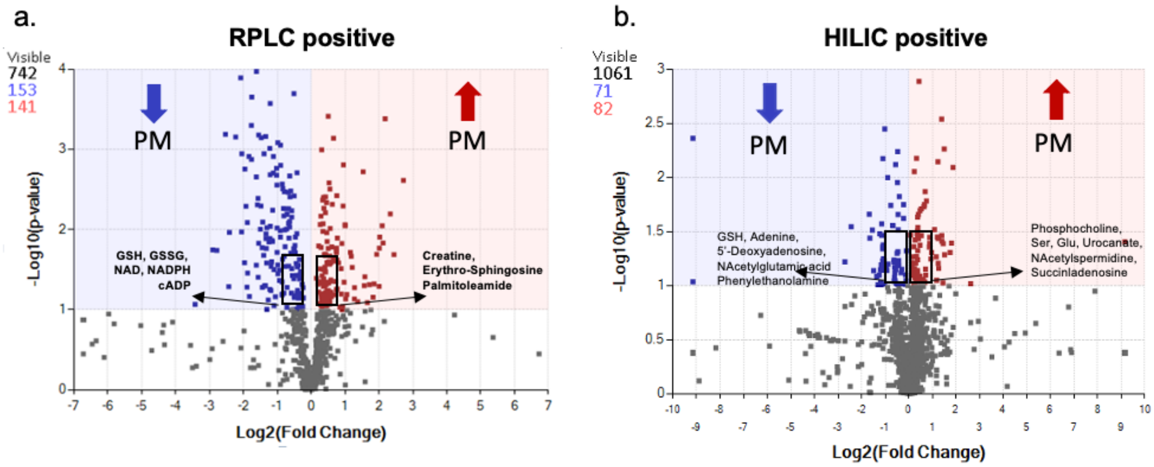

Supplementary Figure 4. The metabolome of SM and PM neural precursors show significant differences. The metabolome of SM and PM neural precursors were analyzed by RPLC positive mode (a) and HILIC positive mode (b) mass-spectroscopy. The total number of compounds (CV<30%) detected were 742 (RPLC) and 1061 (HILIC) of which 294 (RPLC) and 153 (HILIC) are significantly different between PM vs SM. Volcano plots illustrate the total number of detected metabolites (gray) with significantly increased (red) or decreased (blue) metabolites in PM when compared to SM ($p < 0.1$). Representative members of the identified pathways altered between the two brothers are highlighted. Some of the most significantly changed molecules cannot not be annotated, because there are too many isomers as possible candidate metabolites.
